# Dr. John Allen

**Published:** 1892-04

**Authors:** 


### DR. JOHN ALLEN.

THE death of Dr. John Allen, on March 8, in his eighty-second year, marks the passing away from earth of one who has left a positive impression on the dental profession. His record belongs mainly to the past, but its tracings are part of the history of dentistry.

Dr. Allen was born in Broome County, N. Y., November 4, 1810.

He was a descendant of Ethan Allen, of Vermont, famous in Revolutionary annals. He became the student of Dr. James Harris, in Ohio, at the age of nineteen. At the close of his pupilage he commenced his professional career in Cincinnati in 1830.

He secured a knowledge of the manufacture of porcelain teeth, and thus laid the foundation for his success in the production of what was subsequently known as "continuous gum."

This was his most important contribution to dentistry. Others had preceded him in this investigation, but he gave the first practical formula, and cleared away many of the difficulties incident to all new inventions.

He began this work in 1846–47, and filed a caveat in the Patent Office in 1851. This was for a "fusible cement of which an artificial gum is formed applicable to artificial teeth, and by means of which they are set."

He exhibited specimens of this at the American Society of Dental Surgeons. In 1851 a patent was issued, and subsequently the contest between himself and Dr. William Hunter began, to prove priority of invention. This excited intense interest in the dental profession, and until it was settled, in 1855, by compromise, was a disturbing element.

The American Society of Dental Surgeons awarded him a gold medal in 1845, "for his invention for restoring the contour of the face."

The controversy with Dr. Hunter was one of the earliest of the patent contentions, and was practically the basis of the subsequent opposition to dental patents, culminating in the present Dental Protective Association.

The difficulties attending the introduction of continuous gum, and its original imperfect character, made its use very limited; but Dr. Allen persevered, and so far perfected it, through the introduction of platinum as a base, that it eventually became, in the estimation of many, the most satisfactory and artistic of all artificial dentures.

Dr. Allen filled a professorship for several years in the Ohio College of Dental Surgery. This he resigned on his removal to New York City in 1853.

He was interested in the establishment of the New York College of Dentistry, and remained a member of the Board of Trustees up to the time of his death.

In former years he took an active part in the proceedings of the different dental societies, rarely being absent from the sessions of the American Dental Convention and American Dental Association. It was mainly through the efforts of the late Dr. Ambler, of New York, and Dr. Allen that the American Dental Convention continued its moribund career after the formation of the American Dental Association. On the death of the former it ceased to exist as an organization.

Friday, February 19, he performed his last operation in dentistry. Going home that evening, he complained of not feeling well, and from that time he grew gradually weaker until his death.

His funeral took place at his residence, Plainfield, N. J., March 10, 1892.

